# Influence of Dairy Products on Bioavailability of Zinc from Other Food Products: A Review of Complementarity at a Meal Level

**DOI:** 10.3390/nu13124253

**Published:** 2021-11-26

**Authors:** Blerina Shkembi, Thom Huppertz

**Affiliations:** 1 Food Quality & Design Group, Wageningen University & Research, 6708 PB Wageningen, The Netherlands; blerina.shkembi@wur.nl; 2 FrieslandCampina, 3818 LE Amersfoort, The Netherlands

**Keywords:** zinc, zinc absorption, dairy product, meals, co-ingestion, phytate, citrate, phosphopeptides, human, isotope

## Abstract

In this paper, we reviewed the role of dairy products in dietary zinc absorption. Dairy products can have a reasonable contribution for dietary zinc intake in Western diets, where dairy consumption is high. However, the co-ingestion of dairy products can also improve zinc absorption from other food products. Such improvements have been observed when dairy products (e.g., milk or yoghurt) were ingested together with food such as rice, tortillas or bread products, all of which are considered to be high-phytate foods with low inherent zinc absorption. For foods low in phytate, the co-ingestion of dairy products did not improve zinc absorption. Improved zinc absorption of zinc from high-phytate foods following co-ingestion with dairy products may be related to the beneficial effects of the citrate and phosphopeptides present in dairy products. Considering that the main dietary zinc sources in areas in the world where zinc deficiency is most prevalent are typically high in phytate, the inclusion of dairy products in meals may be a viable dietary strategy to improve zinc absorption.

## 1. Introduction

While the nutrient content of food products is typically determined on an individual product basis, the nutritional value is, in many, cases difficult to assess on a product basis. This is because in most cases, food products are consumed as meals rather than as single products. As a result, while the nutrient content of the meal can be considered as the sum of the nutrients in the products making up the meal, this is not true for its nutritional value. The combination of different products into a meal can both improve but also reduce its nutritional value. Examples of (non-)complementary different food products can be observed when considering the proteins in different food products [1]. The nutritional value of proteins is typically expressed based on amino acid composition and the digestibility of the proteins, i.e., proteins that deliver high levels of each of the indispensable amino acids (IAA) in digestible form are scored higher [2]. The lack of one or more IAA in a protein source can result in a lower score for proteins [3]. Such is the case for wheat protein, where the IAA Lys is present only at comparatively low levels [4]. However, some other proteins, including milk proteins, contain Lys in digestible form at levels in excess of those required based on the protein reference patterns [5]. Therefore, the combined consumption of milk protein and wheat protein, e.g., in the form of a breakfast cereal consumed with milk, can create a situation where one protein source, e.g., milk protein, compensates for the deficiencies in another protein sources, e.g., wheat protein [6].

There are, however, instances where the combination of two food sources impairs the digestibility of proteins. This has, for instance, been observed in the case of egg protein, which, when combined with black tea, was shown to have lower digestibility than without black tea [7]. This effect was related to the interaction of polyphenols from the tea with the protein, resulting in impaired digestibility [7,8]. In addition to proteins, the bioavailability of several other nutrients is also affected by co-ingestion with other products, including minerals. In this case, the combination of different products and their mixing in a bolus in the gastro-intestinal tract can lead to the interaction of minerals from one product with constituents from another product. Hurrell et al. [9], for instance, showed that beverages containing polyphenols, such as tea and cocoa could strongly impair the absorption of (non-heme) iron. Likewise, it is also well established that high phytate diets can lead to a reduced absorption of essential minerals such as iron, zinc and calcium, even if they are not present in the same food product [10,11]. Milk and dairy products are found to be an important source of these dietary minerals, supplying more than 50% of dietary calcium in many regions across the world [12,13], but for iron and zinc, milk and dairy products are not the most suitable dietary sources as concentrations of iron and zinc in these products are rather low. However, the consumption of other foods that act as dietary sources of these minerals together with milk and dairy products has been shown to impact absorption. In this paper, we review the impact of dairy products on zinc absorption from other food products. We will consider the physicochemical reasons for the observed in vivo effects, so as to provide a mechanistic understanding of these interactive effects with regard to the meal level. Dietary zinc supplements are not within the scope of this review.

## 2. Zinc in Human Nutrition

Zinc is an essential element because it is required in several cellular processes, such as differentiation, apoptosis and proliferation, which affect growth and development [14]. The adult human body contains ~2–3 g of zinc and most of this zinc is found in the bones and skeletal muscles (>85%), although smaller portions are found in the skin (~4%) and liver (~3%) [15]; the prostate gland contains the highest concentrations of zinc of any soft tissue in the human body [16]. Plasma zinc concentration in healthy individuals range from 12 to 16 μM [17,18,19], but this can be lower during acute infections and inflammation, due the redistribution of zinc from the plasma to the liver [20]. Approximately 60% of plasmatic zinc is associated with albumin, 30% with α-macroglobulin, and the rest with transferrin [21]. Zinc deficiency in humans was first reported in 1961 by Prasad et al. [22], followed by many subsequent reports [23,24], and is currently a worldwide problem that affects ~17% of the global population [25]. South and Southeast Asia, Sub-Saharan Africa and Central America are regions with the highest risk of zinc deficiency [25]. Zinc deficiency can lead to diarrhea, a lack of appetite, hair loss, skin problems, disorders of the gustatory, immunodeficiency and an increased incidence of bacterial, fungal and viral infections [26,27]. Such deficiencies can be related to low intake or the low bioavailability of dietary zinc [28].

Because the human body does not synthesize zinc, the intake of zinc through foods or supplements is required. The population reference intake is 9.4 and 7.5 mg per day for adult (>18 y) males and females, respectively, for those consuming a low phytate diet (300 mg/d), but is notably higher if diets contain more phytate [29]. Among the various food products, red meat, some seafoods, dairy products, nuts, seeds, dried legumes, and whole-grain cereals are considered good dietary sources of zinc [30,31]. For the dietary source of zinc, it is important that these products do not only contain sufficient zinc, but that it is available for absorption. The basic process of zinc absorption is described in Section 3, with the aim of providing the basis for understanding zinc absorption from dairy products and meals containing dairy products, as described in Section 4 and Section 5, respectively.

## 3. Zinc Absorption

The absorption of zinc occurs within the small intestine and the absorbed zinc is released in portal circulation through transporters [32,33,34]. Small intestinal perfusion studies in humans have shown that the sites of maximal absorption of zinc are the duodenum [35] and the jejunum [32]. Zinc is absorbed in its ionized form, so, for the absorption of dietary zinc, it is important that zinc is released from foods and ionized during the digestion process [36]. These liberated zinc ions, however, may form complexes with ligands such as phytates, phosphates, amino acids and other organic acids [34,37,38], which affects their solubility and absorption.

The absorption of zinc in the small intestine occurs by both active (saturable carrier-mediated process) or passive transport (unsaturable diffusion-mediated process) mechanisms, with the former occurring more often at a low luminal zinc concentration and the latter at high concentrations [39]. An active transport of zinc occurs through the following two carriers: ZIP proteins regulate the influx of zinc into enterocytes from the lumen, serum or intracellular compartments, whereas ZnT proteins facilitate the efflux of zinc from the enterocytes into the extracellular environment or into intracellular organelles [39]. Both ZIP and ZnT transporters are present across the entire the gastrointestinal tract, and also in the colon [40]. Zinc exported into the portal vein binds to albumin (60%), with α-macroglobulin (30%), and transferrin [21] for transport to the peripheral tissues. Zinc accumulated in the intestinal epithelial cells can be excreted through their desquamation [41,42].

In general, ~33% of dietary zinc is absorbed in humans [43,44], but this depends on the state of the foods consumed. Zinc absorption from liquid foods is higher (60–70% in fasting subjects) than from solid foods [45]. The amount of zinc in a meal will, in itself, affect zinc absorption: increasing zinc intake from a meal decreases fractional zinc absorption [28,46], which is probably due to the saturation of zinc transporters [47].

## 4. Zinc Absorption from Dairy Products

In countries with a high dairy intake, dairy products contribute significantly to overall zinc intake. For instance, in the Netherlands, >20% of dietary zinc is supplied by dairy products [48]. Dairy consumption provides ~15% of total energy intake, so dairy products proportionally contribute more to zinc intake than to caloric intake [48]. Bovine milk contains ~3–4 mg/L of zinc, of which 95% is associated with the casein micelles and the rest is associated with citrate molecules in the aqueous phase [49].

Human studies concerning the absorption of zinc from dairy products are rather scarce. Sandstrom et al. [50], investigated the absorption of zinc from 450 mL of human milk (casein-whey protein ratio, 40:60), bovine milk with 3% fat (casein-whey protein ratio, 80:20), humanized bovine milk formula (casein-whey protein ratio, 40:60) and soy formula in 54 healthy human adults. Milks and formulas were extrinsically labeled with a radioisotope (^65^Zn) and their absorption was determined by measuring the whole-body retention of the isotope. Even though the zinc content of the formulas was similar to, or higher, than that of human milk, zinc absorption from the formulas and bovine milk was found to be significantly lower (*p* < 0.05) than from human milk (Table 1) [50].

The greater bioavailability of zinc from human milk compared to bovine milk was hypothesized to due to the different chemical composition of the two milks, particularly the different casein:whey protein ratio [50]. This hypothesis based on the importance of the casein:whey protein ratio was later substantiated by findings that proved that adjusting the ratio of casein to whey protein from 80:20 to 40:60 (similar to human milk) significantly (*p* < 0.001) increased zinc absorption from bovine milk, i.e., from 21% to 32% [51]. Moreover, Sandstrom et al. [50] reported a significantly (*p* < 0.05) higher zinc absorption from bovine milk compared to a soy formula (28 vs. 14%) that was high in phytic acid (200 mg/L), which is agrees with the understanding that phytates are powerful inhibitors of zinc uptake [15]. Talsma et al. [52], using a dual stable isotope technique, found lower zinc absorption from bovine milk compared to water (25.5 vs. 72.3%). This is probably because water does not contain inhibitory factors that reduce zinc absorption [53].

## 5. Zinc Absorption from Meals Containing Dairy Products

As outlined in Section 4, the contribution of dairy to total dietary zinc intake is not the highest of all food groups. However, dairy products can still have a notable contribution. In addition, dairy products can play another role in helping to combat zinc deficiency, which is most common in low- and middle-income countries, where the main dietary zinc sources are plant foods, from which zinc is poorly bioavailable [54]. One pathway to improve the absorption of zinc from foods in which zinc is poorly bioavailable, such as cereals and legumes, is the combined intake of these foods together with dairy products. Table 2 shows results of published studies in humans that investigated the complementarity of dairy products for zinc absorption from other products. In these studies, zinc absorption was determined in vivo by isotope techniques.

Talsma et al. [52] examined the effect of milk on zinc absorption from rice in 18 young and healthy women using a dual stable isotopes technique and found a significant (*p* < 0.05) increase (by 62%) in zinc absorption from rice when 90 g of high-phytate rice was consumed together with 600 mL of UHT-treated whole milk, compared to when the same amount of rice was consumed with water. Rosado et al. [55] studied zinc absorption in 48 healthy Mexican women, using a stable double isotope technique, and observed that the addition of 250 mL of UHT low-fat milk or 150 g of flavored yogurt to the plant-based test meal, consisting of 120 g of corn tortillas and 200 g of cooked black beans, significantly (*p* < 0.05) increased zinc absorption by approximately 50% for milk and 68% yoghurt. Sandstrom et al. [56] reported a significant (*p* < 0.001) increase in the zinc absorption from whole meal bread with a high concentration of phytates when ingested with milk and cheese, thereby counteracting the negative effect of phytates and facilitating the absorption of zinc. Using a radioisotope technique, Hunt et al. [57] showed that increasing the amount of casein (8.9 and 51.7 g) in a cereal-based meal, containing rolls made from wholemeal or white flour, significantly (*p* < 0.0001) increased the absorption of zinc in adults. From these studies, it thus appears that the consumption of dairy products in conjunction with food products rich in phytate, i.e., rice [52], corn tortillas [55] or bread [56,57] could increase zinc absorption. However, there are also some reported cases wherein the dietary inclusion of dairy products did not improve zinc absorption, as described below.

Pecoud et al. [60] administered 50 mg of zinc (as 220 mg ZnSO_4_ in gelatin capsules) to 18 subjects orally with breakfast and found that the consumption of 200 mL of milk and 50 g of cheese with breakfast (100 mL coffee, 50 g jam, 10 g butter and 100 g brown bread or 10 g white bread) reduced zinc absorption. It should be noted here, however, that the zinc was provided as a supplement and not via a food product. Sandström and Cederblad [46] reported a significant decrease (*p* < 0.05) in zinc absorption from a soybean meal (consisting of soybeans, potatoes, tomatoes and white bread) by the addition of milk (125 mL) and hypothesized that the additional calcium provided by milk could hinder zinc absorption. A non-significant (*p* > 0.05) reduction in zinc absorption was observed by Flanagan et al. [59] when 210 mL of milk (made up to 250 mL with water) was provided with a turkey-based meal, compared when the meal was consumed with water. Wood and Zheng [60] also found no significant effect (*p* > 0.05) on zinc absorption when 400 mL of reconstituted non-fat dried milk per day (200 mL with breakfast and 200 mL with dinner) was consumed with basal diets consisting of normal foods, selected to provide the U.S. Recommended Dietary Allowances. Contrary to earlier studies where dairy products improved zinc absorption from primarily phytate-rich foods, it appears that for other foods this was not that case. As outlined in Table 2, the fractional absorption of zinc from the base meal without dairy was notably higher (19–29%) than of that in studies with phytate rich foods (7–13%). It thus appears that the ability of dairy products to improve zinc absorption is dependent on a product matrix, which is further described in Section 6.

## 6. Factors Affecting Zinc Absorption from Meals Containing Dairy Products

From Table 2, it is apparent that, cases where the consumption of dairy products in a meal improved zinc absorption from other products, are cases where the dietary zinc was provided by phytate-rich foods (e.g., rice, bread, tortilla). When analyzing the role of dairy mechanistically, it is important to consider that milk and dairy products contain various compounds that also have the ability to bind zinc, e.g., citrate, phosphate and proteins. Two studies have reported that the absorption of zinc is strongly related to the solubility of zinc salts in an aqueous solution [61,62]. Tang and Skibsted [63] compared the solubility of zinc phytate and zinc citrate salts in water at ~37 °C and noted that the former had a solubility that was ~5 orders of magnitude lower (2.9 × 10^−6^ vs. 0.32 g/100 mL), a finding that was also observed in concentrations of zinc ions in solution (1.6 × 10^−7^ vs. 0.017 mol/L). Dietary zinc can interact with dietary phytate in the intestinal lumen (luminal pH 6–7.4) [64], forming stable and sparingly soluble complexes which precipitate, and thus become poorly bioaccessible [28]. The precipitated zinc-phytate complexes are therefore excreted with the feces [15]. In other words, dietary phytate interferes with zinc uptake and is therefore considered to be one of the main inhibitors of zinc absorption. According to the World Health Organization [65] a phytate:zinc molar ratio of higher than 15:1 inhibits zinc bioavailability.

The stability and solubility of the zinc/phytate complex depends on various factors such as the pH value, the molar ratio and the presence of other compounds in the solution [66]. Phytate binds zinc with a stoichiometric ratio of 2:1 of the zinc/phytate complex [15] with strong binding affinities, as follows: 1.8 × 10^6^ L/mol (site 1) and 8 × 10^4^ L/mol (site 2) [63]. On the other hand, citrate binds to zinc with a weaker strength (1.2 × 10^4^ L/mol) than phytate. Citrate is the main low molecular weight zinc ligand in human milk and is known as a zinc absorption enhancer [15,28]. Human and bovine milk contains 80 mg/100 mL and 150 mg/100 mL of citrate, respectively [67], which can help to solubilize better zinc from other foods, providing that they are consumed at the same time [68]. As a result, the bioavailability of zinc as a result of combining milk and other dairy products with foods may be considerably higher than that of the foods themselves.

Several hypotheses have been posed for improved zinc absorption from phytate-rich foods when consumed in combination with dairy products. It has been hypothesized that this improvement in zinc absorption from phytate-rich foods by co-ingesting dairy products may be due to a higher proportion of phytate-bound calcium in the gastrointestinal tract, thereby blocking phytate binding sites and allowing more zinc to become available for absorption [51]. However, Hansen et al. [69] observed no significant effect (*p* > 0.05) on zinc absorption after the addition of a high amount of calcium to high phytate meals. Several studies reported that a phytate x Ca:Zn molar ratio above 200 is needed to hamper zinc absorption [70,71].

One suggested explanation for the improvement of zinc absorption by dairy products is that amino acids (e.g., histidine, methionine) or peptides, that become liberated during the digestion of milk proteins, bind zinc in the intestinal lumen at intestinal pH and form complexes, thereby increasing both their bioavailability and solubility [28,72]. These complexes are probably absorbed in the brush border membrane of enterocytes through amino acid transporters [47].

Other studies suggest that the increase of zinc absorption by dairy products could be due to casein phosphopeptides (CPPs), which are phosphorylated peptides that are released when casein is digested and can bind zinc, thereby preventing it from binding to phytate [28,46,73]. Reynolds [74] reported that one CPP molecule can chelate up to six zinc ions. Hansen et al. [75] found a significantly higher (*p* < 0.05) zinc absorption in humans after the addition of CPPs to a rice-based cereal (composition: rice flour, skimmed milk powder, maltodextrin, vegetable fat, and dry ground peas, carrots, cauliflower, broccoli, and leek) that was low in phytate (20 mg per serving), but no significant effect (*p* > 0.05) on zinc absorption occurred when CPPs were added to whole grain cereal (composition: rice, wheat, corn, oats, skimmed milk powder, lactose, vegetable fat, oat bran, maltodextrin) that were high in phytate (198 mg per serving). Hansen et al. [69] found no significant effect (*p* > 0.05) on zinc absorption after the addition of different amounts of CPP to bread-based meals (consisting of a roll served with 15 g of jam, 8 g of butter and 250 mL of water) (Table 3). Three different bread rolls were formulated: high-phytate/high calcium, high-phytate/low calcium, low-phytate/high calcium; however, the authors indicate that even the “low-phytate/high calcium” rolls contained a rather high amount of phytate (~101 mg) [69]. These results, taken together, suggest that the effect of CPPs on zinc absorption may depend on the phytate content of the meal. In particular, with foods containing relatively low amounts of phytates, CPPs have a positive effect on zinc absorption, while with foods relatively rich in phytates, a positive effect has not been observed. Higher doses of CPPs are likely required for zinc to be released from the phytate complex [69]. Hansen et al. [76] observed an improvement in zinc absorption in rat pups by adding CPPs to a phytate-containing meal (~264 mg phytate/L). In the latter study, the dose of CPPs was higher than in previously reported human studies [69,75]. Furthermore, unlike human studies, CPPs were added to an aqueous solution and were not administered in a solid meal. It is reasonable to hypothesize that the food matrix may also have positively influenced the effect of CPPs on zinc absorption. It is well known that zinc is more easily absorbed from aqueous solutions than from solid foods [28]; moreover, CPPs may be better absorbed by a liquid matrix [73].

## 7. Conclusions

From the results explored above, it is clear that dairy products play a dual role with regard to the absorption of dietary zinc. Both provide a source of dietary zinc, which in general has good bioavailability. However, in addition to this, they also modulate the absorption of zinc from other food sources. Such effects are arguably not unique to only dairy products or zinc, and warrant further investigation and the consideration of the bioavailability of nutrients at a meal level rather than an individual product level. Considering that areas where zinc deficiencies are most prominent are also those where zinc is often ingested via high phytate products, dietary recommendations on the inclusion of dairy products, or products with a comparable ability to modulate zinc absorption in meals, can present significant advantages.

## Figures and Tables

**Table 1:** Fractional zinc absorption in humans from milk and dairy products.

Meals	Zinc Intake (mg)	Zinc Absorption (%) ^1^	Significance	Methods	References
450 mL Human Milk (Casein:Whey Ratio, 40:60) (*Control*)	1.3	41.0 ± 2.3			
450 mL Bovine Milk, 3% Fat (Casein:Whey Ratio, 80:20)	1.6	28.0 ± 6.1	S	Radioisotope	[50]
450 mL Humanized Bovine Milk Formula (Casein:Whey Ratio, 40:60)	1.2	31.0 ± 1.8	S		
450 mL soy Protein-Isolate Formula	1.7	14.0 ± 1.4	S		
450 mL Bovine Milk Formula (Whey:Casein 60:40) (*Control*)	1.2	32.2 ± 1.4			
450 mL Bovine Milk Formula (Whey:Casein 20:80)	3.2	21.3 ± 2.9	S	Radioisotope	[51]
Water (*Control*)	4.22	72.3 ± 1.7		Dual stable isotopes	[52]
Bovine Milk	4.04	25.5 ± 1.8	S		

^1^ Values are mean estimates ± SEM S, significant difference vs. 1 the control sample in the study, as highlighted in the first row for each study (for more information see text).

**Table 2:** Influence of the inclusion of milk and dairy products to meals on zinc absorption from such meals in humans.

Meals	Zinc Intake (mg)	Zinc Absorption (%) ^1^	Significance	Methods	References
90 g Cooked Rice + 600 mL Water (*Control*)	3.8	12.8 ± 0.9		Dual stable isotopes	[52]
90 g Cooked Rice + 600 mL Milk full Fat UHT	3.6	20.8 ± 0.9	S		
Plant-Based Test Meal (*Control*)	4.8	7.1 ± 1.2			
Plant-Based Test Meal + 250 mL of Milk	5.8	10.6 ± 1.2	S	Dual stable isotopes	[55]
Plant-Based Test Meal + 150 g of Yogurt	5.7	11.9 ± 1.2	S		
60 g Wholemeal Bread (*Control*)	3.5	8.2 (5.7–11.3)			
60 g Wholemeal Bread + 200 g Milk	3.1	9.9 (5.6–14.4)		Radioisotope	[56]
60 g Wholemeal Bread + 200 g Milk + 42 g Cheese	3.2	14 (8.7–21.8)	S		
Rolls with White Flour + 8.9 g Casein (*Control*)	4.1	13.0 ± 0.6			
Rolls with White Flour + 50.5 g Casein	3.9	26.0 ± 2.2	S	Radioisotope	[57]
Rolls with Whole Wheat Flour + 10.6 g Casein (*Control*)	3.9	8.0 ± 1.3			
Rolls with Whole-Wheat Flour + 51.7 g Casein	4.0	25.0 ± 2.2	S		
Soybean Meals (*Control*)	2.5	19.6 ± 2.0		Radioisotope	[46]
Soybean Meals + 125 mL Milk	2.7	14.1 ± 0.5	S		
Turkey Meal + 250 mL Deionized Water (*Control*)	4.0	29.0 ± 2.2		Dual isotopes	[58]
Turkey Meal + 210 mL Milk	4.0	22.0 ± 2.5	NS		
Basal Diets (*Control*)	14.5	22.0 ± 6		Dual isotopes	[59]
Basal Diets + 400 mL Milk Per Day	16.0	23.0 ± 6	NS		

^1^ Values are mean estimates ± SEM. NS, non-significant difference vs. control the control sample in the study, as highlighted in the first column (for more information see text). S, significant difference vs. the control sample in the study, as highlighted in the first row for each study (for more information see text). Basal diets, normal foods chosen to provide the U.S. Recommended Dietary Allowances.

**Table 3:** Influence of calcium, casein phosphopeptides (CCP) and phytates on zinc absorption.

Meals	Zinc Intake (mg)	Zinc Absorption (%) ^1^	Significance	Methods	References
Rice-Based Cereal + 0 g CPP	1.29	19.4 ± 2.7			
Rice-Based Cereal + 1 g CPP	1.29	25.2 ± 2.3	S		
Rice-Based Cereal + 2 g CPP	1.29	23.9 ± 1.6	S	Radioisotope	[75]
Whole Grain Cereal + 0 g CPP	1.77	16.0 ± 1.5			
Whole Grain Cereal + 1 g CPP	1.77	15.3 ± 0.9	NS	Radioisotope	[75]
Whole Grain Cereal + 2 g CPP	1.77	18.1 ± 1.3	NS		
Bread Meal, High-Phytate/High Calcium + 0 mg CPP	1.4	7.0 ± 0.5			
Bread Meal, High-Phytate/High Calcium + 250 mg CPP	1.4	7.7 ± 0.9	NS	Radioisotope	[75]
Bread Meal, High-Phytate/High Calcium + 1000 mg CPP	1.4	8.0 ± 0.8	NS		
Bread Meal, High-Phytate/Low Calcium + 0 mg CPP	1.3	7.7 ± 0.8			
Bread Meal, High-Phytate/Low Calcium + 250 mg CPP	1.3	7.0 ± 0.7	NS	Radioisotope	[69]
Bread Meal, High-Phytate/Low Calcium + 1000 mg CPP	1.3	6.5 ± 0.5	NS		
Bread Meal, Low-Phytate/High Calcium + 0 mg CPP	1.5	14.3 ± 1.4			
Bread Meal, Low-Phytate/High Calcium + 250 mg CPP	1.5	16.7 ± 2.1	NS	Radioisotope	[69]
Bread Meal, Low-Phytate/High Calcium + 1000 mg CPP	1.5	16.0 ± 2.8	NS		

^1^ Values are mean estimates ± SEM. NS, non-significant difference vs. the sample with no CPP added (for more information see text). S, significant difference vs. the sample with no CPP added (for more information see text).
